# Utility of Artificial Intelligence in Detecting Lymph Node Metastasis in Oral Cancer: A Systematic Review

**DOI:** 10.7759/cureus.116745

**Published:** 2026-09-22

**Authors:** Mrunali G Gharat, Sneha Masne Deshpande, Jyotsna Sethumadhavan, Prachi Gholap, Swati Kale, Sheetal Choudhari, Ameya P More

**Affiliations:** 1 Dentistry, Bharati Vidyapeeth (Deemed to be University) Dental College and Hospital, Navi Mumbai, IND; 2 Oral Pathology and Microbiology, Bharati Vidyapeeth (Deemed to be University) Dental College and Hospital, Navi Mumbai, IND; 3 Prosthodontics and Crown &amp; Bridge, Bharati Vidyapeeth (Deemed to be University) Dental College and Hospital, Navi Mumbai, IND; 4 Prosthodontics, Bharati Vidyapeeth (Deemed to be University) Dental College and Hospital, Navi Mumbai, IND; 5 Pediatric and Preventive Dentistry, Bharati Vidyapeeth (Deemed to be University) Dental College and Hospital, Navi Mumbai, IND; 6 Oral Pathology and Microbiology, Dr. G.D. Pol Foundation Y.M.T. Dental College and Hospital, Navi Mumbai, IND; 7 Periodontology, Bharati Vidyapeeth (Deemed to be University) Dental College and Hospital, Navi Mumbai, IND

**Keywords:** artificial intelligence, deep learning, diagnostic accuracy studies, lymph node metastasis, machine learning, occult metastasis, oral cancer, oral squamous cell carcinoma

## Abstract

Oral squamous cell carcinoma (OSCC) is associated with considerable morbidity because of occult lymph node metastasis (OLNM). Conventional imaging has limitations in detecting OLNM in oral cancer. AI-based technologies have been proposed as potential aids in diagnosis. This systematic review was carried out following PRISMA 2020 guidelines and registered in the International Prospective Register of Systematic Reviews (PROSPERO) (February 2025). PubMed, Scopus, Embase, Web of Science, and Google Scholar were searched. Studies utilizing artificial intelligence (AI)-based techniques, such as machine learning (ML), deep learning (DL), and convolutional neural networks (CNNs), in the detection of lymph node metastasis in oral cancer with reported results of diagnostic accuracy, sensitivity, specificity, or area under the curve (AUC) were included. Methodological quality was assessed by Quality Assessment of Diagnostic Accuracy Studies 2 (QUADAS-2); evidence certainty was assessed by Grading of Recommendations, Assessment, Development, and Evaluation (GRADE). A total of 15 articles (n = 5,758) fulfilled the inclusion criteria. The AI-based models yielded AUC values of 0.729-0.961, sensitivity of 0.65-1.00, and specificity of 0.576-0.98. DL-based CNN architectures and radiomics-DL hybrid models showed the highest diagnostic accuracy. Moderate overall risk of bias was reported with the QUADAS-2 tool, while GRADE provided certainty of evidence as low to very low due to study heterogeneity and lack of external validation. The techniques employing AI appear to have great potential for diagnosing OLNM in patients with mouth cancer because they have more accurate capabilities than imaging. There is a need for future multicenter validation, standardization of algorithms, and ethical frameworks for their application.

## Introduction and background

Cancer is an important global disease, with 20 million deaths from this cause reported globally in 2022 [1]. Oral cancer (OC), especially oral squamous cell carcinoma (OSCC), is a significant public health issue. It ranks sixth as a cause of cancer worldwide, when grouped together with pharyngeal cancers. There are about 275,000 newly diagnosed cases each year, of which two-thirds come from developing nations [2].

OSCC is characterized by major differences in occurrence among populations and geographical areas, increased susceptibility to second malignancies, and a continuing poor prognosis with a five-year survival rate of about 50%, mainly because of late-stage diagnosis [3,4]. Metastasis remains the leading cause of cancer-related mortality, accounting for approximately 90% of cancer deaths, with lymph node (LN) metastasis in the cervical region representing the most critical prognostic factor in OSCC [5,6].

Occult lymph node metastasis (OLNM) refers to the presence of cancer cells in regional LNs undetectable by conventional clinical or radiological assessment. The incidence of OLNM in clinically node-negative (cN0) patients is reported at up to 26.4% [7]. Detection failure results in significant under-treatment and adversely affects prognosis. Conventional imaging modalities, including computed tomography (CT) and magnetic resonance imaging (MRI), demonstrate sensitivity as low as 35.7% for identifying OLNM, while sentinel lymph node biopsy (SLNB) has shown 71.4% sensitivity in early-stage OSCC [8].

Artificial intelligence (AI), which includes machine learning (ML), deep learning (DL), and convolutional neural networks (CNNs), has shown its ability to transform oncological imaging and diagnostics [9]. AI systems have been found to recognize patterns in imaging data that cannot be detected with the human eye, providing better sensitivity and specificity [10]. Radiomics, which entails the extraction of quantitative features from imaging data using ML/DL techniques, has further enhanced the applications of AI in head and neck oncology [11,12]. The advent of explainable AI (XAI) has enabled clinicians to gain an understanding of the workings of the models used [13].

Accurate detection of OLNM is particularly important in cN0 oral cancer, where clinicians must balance elective neck dissection against close surveillance. AI may support this decision by identifying subtle patterns within clinical and imaging data. ML and DL, including CNNs, enable automated pattern recognition, while radiomics extracts quantitative imaging features and XAI improves the interpretability of model predictions. These approaches may therefore enhance the preoperative identification of occult nodal disease and assist risk stratification in cN0 patients.

In spite of accumulating data from individual studies, a systematic synthesis of the effectiveness of AI for detecting OLNM in OC is still needed. While previous systematic reviews have explored the use of AI in the field of head and neck cancers [14,15] or just prognosis [16], no systematic study has yet been conducted on the diagnosis of AI for OLNM in OC through quality assessment. The objectives of the current systematic review include the following: (1) assessment of the performance of AI in detecting OLNM in OC; (2) comparison between AI-assisted methods and conventional imaging and histopathological methods; (3) clinical implications of AI for treatment planning; and (4) identification of present limitations and future directions.

## Review

Methods

The present systematic review was conducted and reported following the guidelines of the Preferred Reporting Items for Systematic Reviews and Meta-Analyses (PRISMA) 2020 statement (Appendices) [17]. The protocol was pre-registered in the International Prospective Register of Systematic Reviews (PROSPERO) on 18th February 2025 (Registration Number: CRD42025638435). The literature search in PubMed, Scopus, Embase, Web of Science, and Google Scholar was conducted up to the date of the search. The search strategy used MeSH and free-text keywords including the following: ("Artificial intelligence" OR "Machine learning" OR "Deep learning" OR "Convolutional neural network" OR "Radiomics") AND ("Lymph node metastasis" OR "Metastatic lymph nodes" OR "Occult metastasis" OR "Cervical lymph node") AND ("Oral cancer" OR "Oral squamous cell carcinoma" OR "OSCC" OR "Oral cavity cancer") AND ("Diagnostic accuracy" OR "Prediction model" OR "AUC" OR "Sensitivity" OR "Specificity"). No language or date restrictions were applied during the search. Reference lists of included studies and relevant systematic reviews were additionally searched.

Studies were eligible for inclusion if they met the following population, index test, comparison, and outcome (PICO)-aligned criteria. Population: adult patients (≥18 years) diagnosed with oral cancer or OSCC, including those cN0. Index test: AI-based approaches including ML, DL, CNN, or radiomics models for detection or prediction of lymph node metastasis (LNM) or OLNM. Comparator: conventional imaging (CT, MRI, PET), clinical assessment, or histopathological examination. Outcomes: at least one diagnostic performance metric reported - accuracy, sensitivity, specificity, or area under the curve (AUC).

Eligible study designs included prospective or retrospective cohort studies, case-control studies, or randomized controlled trials published in English. Studies were excluded if they evaluated AI for non-oral cancer populations; used non-human or in vitro models; were narrative reviews, editorials, case reports, or conference abstracts without peer-reviewed full-text; or reported insufficient diagnostic data precluding extraction of performance metrics. No restriction on sample size was applied. Two independent reviewers (MG and SM) screened all titles and abstracts retrieved from database searches, followed by full-text assessment of potentially eligible studies. Disagreements were resolved by consensus or by consultation with a third reviewer (JS). Inter-rater agreement was calculated using Cohen's kappa coefficient [18].

The methodological quality of the included studies was evaluated via Quality Assessment of Diagnostic Accuracy Studies 2 (QUADAS-2) (Table 1) [19]. QUADAS-2 risk of bias assessment of all 15 included studies was conducted in four areas, namely, (1) patient selection, (2) index test, (3) reference standard, and (4) flow and timing. All of the above-mentioned areas were rated as low, high, or unclear risk of bias. Assessments were done independently by two reviewers, and any disagreement between the two was settled via discussion. Data extraction was done by one reviewer (MG) independently and validated by another reviewer (SM) using a pre-piloted standardized data extraction form. Variables that were extracted included the following: study characteristics (author(s), year, country, study design); patient characteristics (sample size, age, sex, cancer stage); details about the AI model (type of algorithm, imaging modality, training/validation); and diagnostic accuracy (accuracy, sensitivity, specificity, AUC).

**Table 1:** QUADAS-2 risk of bias assessment of all 15 included studies. QUADAS-2: Quality Assessment of Diagnostic Accuracy Studies 2; RoB = risk of bias.

Study	Patient selection	Index test	Reference standard	Flow and timing	Applicability	Overall RoB
Ariji et al., 2020 [20]	Unclear	Low	Low	Unclear	Low	Unclear
Zhou et al., 2018 [21]	Unclear	Unclear	Low	Unclear	Unclear	Unclear
De Silva et al., 2018 [22]	Low	Low	Low	Low	Low	Low
Bur et al., 2019 [23]	Unclear	Low	Low	Unclear	Low	Unclear
Mermod et al., 2020 [24]	High	Unclear	Low	Unclear	Unclear	High
Romeo et al., 2019 [25]	High	Unclear	Low	Unclear	Unclear	High
Shan et al., 2020 [26]	Unclear	Low	Low	Unclear	Low	Unclear
Tomita et al., 2021 [27]	Unclear	Low	Low	Unclear	Low	Unclear
Ariji et al., 2019 [28]	Unclear	Low	Low	Unclear	Low	Unclear
Farrokhian et al., 2022 [29]	Low	Low	Low	Low	Low	Low
Vidiri et al., 2023 [30]	Unclear	Unclear	Low	Unclear	Unclear	Unclear
Xu et al., 2023 [31]	Unclear	Low	Low	Unclear	Low	Unclear
Chen et al., 2023 [32]	Unclear	Low	Low	Unclear	Low	Unclear
Esce et al., 2024 [33]	Unclear	Unclear	Low	Unclear	Unclear	Unclear
Liu et al., 2024 [34]	Low	Low	Low	Unclear	Low	Low

Synthesis Methods

Due to substantial clinical, methodological, and technical heterogeneity among the included studies, quantitative meta-analysis was not considered appropriate. Therefore, a structured narrative synthesis was performed. Studies were grouped according to AI methodology, imaging modality, study design, and diagnostic outcomes. Reported measures of diagnostic performance, including sensitivity, specificity, accuracy, and AUC, were summarized descriptively and compared across studies, while training and validation results were considered separately where available. The synthesis focused on identifying consistent patterns, methodological differences, and sources of heterogeneity across the available evidence.

The certainty of evidence was assessed using the Grading of Recommendations, Assessment, Development, and Evaluation (GRADE) framework, which is shown in Table 2 [35]. Given the diagnostic accuracy focus of this review, domains evaluated included risk of bias, inconsistency, indirectness, imprecision, and publication bias. A narrative synthesis was conducted given the substantial heterogeneity in AI model types, imaging modalities, patient populations, and outcome reporting across studies, which precluded meta-analysis.

**Table 2:** GRADE certainty of evidence. GRADE: Grading of Recommendations, Assessment, Development, and Evaluation; AUC: area under the curve; OLNM: occult lymph node metastasis; CNN: convolutional neural network; DL: deep learning; RCT: randomized controlled trial; END: elective neck dissection.

Outcome	Studies	Patients	Risk of bias	Inconsistency	Indirectness	Imprecision	Publication bias	Certainty	Summary of findings
Diagnostic accuracy (AUC)	15	5,758	Serious (–1)	Serious (–1)	Not serious	Not serious	Undetected	Low (⊕⊕⊝⊝)	AUC: 0.729–0.961; pooled estimate not calculable due to heterogeneity
Sensitivity for OLNM	13	5,132	Serious (–1)	Serious (–1)	Not serious	Not serious	Undetected	Low (⊕⊕⊝⊝)	Range: 0.65–1.00; high variability by model type and imaging modality
Specificity for OLNM	13	5,132	Serious (–1)	Serious (–1)	Not serious	Not serious	Undetected	Low (⊕⊕⊝⊝)	Range: 0.576–0.98; highest with CNN and hybrid radiomics-DL
AI vs. conventional imaging	6	2,844	Very serious (–2)	Serious (–1)	Not serious	Serious (–1)	Suspected	Very Low (⊕⊝⊝⊝)	AI consistently outperforms CT/MRI alone; no RCT-level comparisons
Clinical impact on neck dissection	4	2,788	Serious (–1)	Not serious	Serious (–1)	Serious (–1)	Undetected	Very Low (⊕⊝⊝⊝)	Potential to reduce unnecessary END; no prospective outcome data

An initial database search identified 170 records; two additional records were retrieved through citation searching (total: 170). Following removal of 47 duplicates, 123 records underwent title and abstract screening, of which 98 were excluded. Full-text assessment was performed on 25 articles, and 10 were excluded as they did not meet the eligibility criteria; these studies are presented in the Appendices. Fifteen studies fulfilled the eligibility criteria and were included in the final synthesis (Figure 1). Inter-rater agreement for study selection was substantial (Cohen's kappa = 0.82).

**Figure 1:**
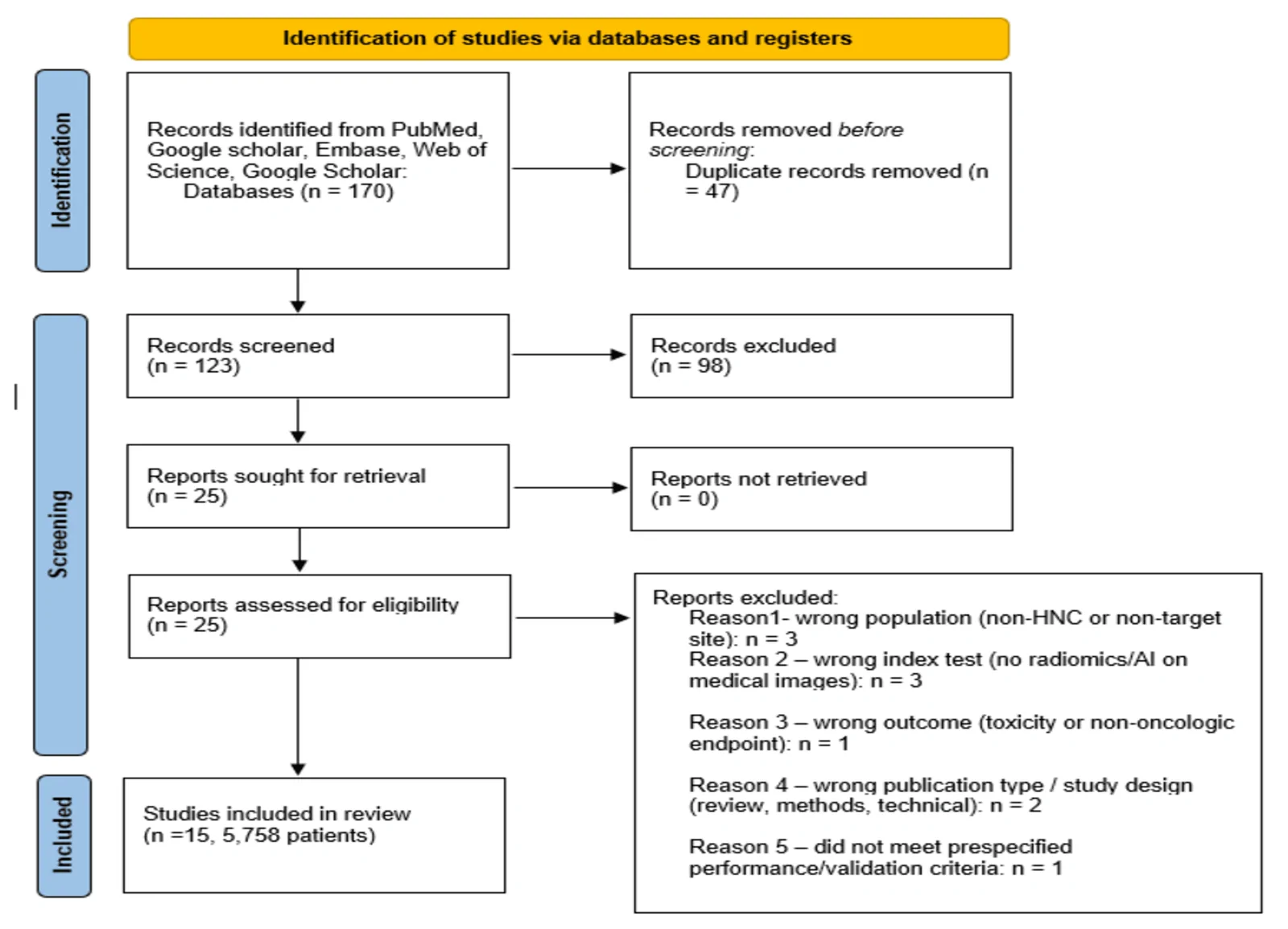
PRISMA 2020 flow diagram illustrating the study selection process. HNC: head and neck cancer.

Results

Across the 15 included studies involving 5,758 patients, AI-based approaches demonstrated variable diagnostic performance for detecting lymph-node metastasis in oral cancer. Reported AUC values ranged from 0.729 to 0.961, while sensitivity ranged from 0.65 to 1.00 and specificity from 0.576 to 0.98. These values represent individual study estimates and were not pooled because of substantial heterogeneity in patient populations, imaging modalities, AI architectures, reference standards, and validation strategies. Training, validation, and test-set results were considered separately where reported. Overall, the available evidence suggested potentially high diagnostic performance of several AI models; however, differences in study design and validation methods limited direct comparison between studies. Detailed study-level diagnostic estimates are presented in Table 3.

**Table 3:** Data extraction table. Author & year column includes reference numbers for each study. NR: not reported; DL: deep learning; ML: machine learning; CNN: convolutional neural network; RF: random forest; SVM: support vector machine; AUC: area under the curve; KNN: k-nearest neighbors; NB: naïve Bayes; OSCC: oral squamous cell carcinoma; OTSCC: oral tongue squamous cell carcinoma; DOI: depth of invasion; LNs: lymph nodes; LNM: lymph node metastasis.

No.	Author & year	Country	Study design	Sample size	Age	AI model	Accuracy	Sensitivity	Specificity	Conclusion
1	Ariji et al., 2020 [20]	Japan	Retrospective	45	Mean 63 years	Deep Learning (Nvidia GeForce GTX)	0.782	0.81	0.754	No significant difference between DL and radiologists
2	Zhou et al., 2018 [21]	China	Predictive model	59	NR	Hybrid Model (MaO-radiomics + 3D-CNN)	0.88	0.95	N/A	Hybrid AI improved prediction using PET & CT
3	De Silva et al., 2018 [22]	New Zealand	Retrospective	623	90% ≥40 years	Simple predictive model	N/A	0.83	0.63	Useful model for developing regions
4	Bur et al., 2019 [23]	USA	Retrospective	1961	NR	Random Forest (RF)	0.84	0.917	0.576	ML improves nodal metastasis prediction
5	Mermod et al., 2020 [24]	Switzerland	Retrospective	168	Train=62.4; Val=61.8 years	Random Forest	0.88	1.00	0.98	AI-driven risk stratification improves OSCC management
6	Romeo et al., 2019 [25]	Italy	Retrospective	40	69±14 years	Radiomic ML (KNN, NB)	≥90%	100% (KNN)	100% (NB)	Radiomic ML predicts tumor grade & nodal status
7	Shan et al., 2020 [26]	China	Cross-sectional	101	~59.8 years	RF, SVM, Naïve Bayes	AUC=0.786	0.85	0.75	ML outperforms conventional predictors
8	Tomita et al., 2021 [27]	Japan	Retrospective	39	64±14 years	Xception-based DL	0.859	0.667	0.954	DL differentiates benign vs. metastatic cervical LNs
9	Ariji et al., 2019 [28]	Japan	Retrospective	59	62.7±11.7 years	CNN U-Net	0.961	0.979	0.953	AI outperformed human evaluation
10	Farrokhian et al., 2022 [29]	USA	Cohort	634	≥18 years	Predictive model (DOI-based)	Higher	0.917	0.726	ML aids in reducing unnecessary neck dissections
11	Vidiri et al., 2023 [30]	Italy	Retrospective	108	NR	MRI-based ML	Train=0.86; Val=0.81	0.73–0.81	0.71–0.73	MRI-driven ML stratifies OTSCC patients
12	Xu et al., 2023 [31]	China	Retrospective	1466	55.4 years	DL segmentation algorithm	0.851	N/A	N/A	AI enhances diagnosis & treatment planning
13	Chen et al., 2023 [32]	China	Retrospective	100	NR	DL + Radiomics fusion model	89.20%	0.92	0.889	Fusion model improves LNM prediction
14	Esce et al., 2024 [33]	USA	Retrospective	89	NR	CNN with HALO-AI	AUC=0.729	0.65	0.86	CNN predicts nodal metastases using histopathology
15	Liu et al., 2024 [34]	China	Retrospective	326	NR	Radiomics-based AI	0.952 & 0.877	0.984 & 0.815	0.93 & 0.933	AI helps guide neck dissection decisions

Discussion

The current systematic review summarizes data from 15 studies investigating the use of AI in detecting OLNM in patients with OC. Overall, the results show that there is great promise in the diagnostic accuracy of AI models, including ML, DL, and CNN models, with AUC ranging up to 0.961, 100% sensitivity, and 98% specificity. From the included studies, the work by Ariji et al. [28], which utilized a CNN U-Net architecture on CT scans, yielded the highest overall accuracy (96.1%), surpassing the performance of radiologists who had undergone training. The capacity for U-Net architecture-based models to segment tumors is especially useful for identifying characteristics of metastatic lymph nodes such as irregular margins, heterogeneous enhancement, and extranodal extension, which are subjective assessments done by clinicians. A study conducted by Mermod et al. [24] established a random forest model having 100% sensitivity and 98% specificity for OLNM diagnosis of early-stage OSCC. Such results far outshine traditional image modality performance, where CT and MRI were found to have a sensitivity rate as low as 35.7% [22]. In the early work by Ariji et al. [20], there was proof that the interpretation of contrast-enhanced CT using AI techniques was on par with and even better than the diagnostic consistency of skilled radiologists.

A hybrid model combining radiomics and a DL framework [33] achieved an accuracy of 89.2%, which proves the ability to enhance the predictive power through multimodal data fusion compared to a single-modality approach [24]. In addition, MRI radiomics models [30,34] proved their efficiency in decision-making about neck dissection of oral tongue squamous cell carcinoma (OTSCC). The ML models evaluated by Bur et al. [23] demonstrated in a large cohort of 1,961 patients that random forest algorithms substantially outperform conventional clinicopathological predictors for nodal metastasis risk. Romeo et al. [25] demonstrated that radiomic ML analysis of MRI-derived texture features could predict tumor grade and nodal status with specificities reaching 100%. Shan et al. [26] further validated that ML-derived feature combinations outperform depth of invasion (DOI) as a standalone predictor, with AUC values of 0.786. Radiomics-based approaches applied to intraoral ultrasound have also shown promise, offering a radiation-free, bedside-accessible modality for predicting occult nodal disease. The Xception-based DL framework of Tomita et al. [27], applied to preoperative CT, achieved 85.9% accuracy with a specificity of 0.954. Xu et al. [31] applied a DL segmentation algorithm to 1,466 patients, establishing that AI-enhanced CT interpretation has direct implications for treatment planning. The present findings are consistent with broader systematic reviews of AI in head and neck oncology [36,37]. PET/CT-based radiomics models have demonstrated additional value for functional characterization of nodal disease [38,39]. A recent meta-analysis by Deng et al. [40] confirmed diagnostic superiority of AI-augmented imaging over conventional assessment, with pooled AUC estimates consistently exceeding 0.85. Vidiri et al. [30] demonstrated that combining tumor stage imaging features with cervical nodal ML signatures yielded classification accuracy superior to either parameter alone. Esce et al. [33] demonstrated that CNN-based analysis of histopathological whole-slide images could predict nodal metastasis with an AUC of 0.729, leveraging routinely obtained diagnostic material without additional imaging. Lan et al. [41] developed a combined DL + radiomics MRI model for simultaneous prediction of occult LNM and prognosis. Gang et al. [42] applied a lightweight MobileNetV2 U-Net architecture combined with multiparametric MRI radiomics, demonstrating practical scalability for clinical deployment.

Another important limitation is geographic clustering; the majority of studies included in this review have come from China, Japan, the USA, and Europe. None of the studies included were performed in South Asia, Sub-Saharan Africa, and Latin America, where there is an overrepresentation of oral cancer burden [43]. This brings up an important issue of algorithmic bias: models trained on a cohort of patients with primarily Western or Eastern Asian genetic background might fail in the real-world setting due to their inability to recognize the differences in biological and etiological factors of oral cancer in various populations.

The use of retrospective single-center studies and internal validation models is a major limitation. External validation, which is a necessary requirement before application in the clinic, is missing in 13 out of 15 studies. It is highly likely that there are overly optimistic performance estimates owing to overfitting. Standardization of imaging acquisition and radiomic feature extraction pipelines is an absolute prerequisite for conducting reproducible research [44]. The interpretability of algorithms is yet another hurdle. Most DL algorithms work as "black box" algorithms. Integration of techniques in XAI, such as SHAP (SHapley Additive exPlanations) and LIME (Local Interpretable Model-agnostic Explanations), and attention map visualization in future studies on oral cancer AI is highly recommended [45].

Ethical issues such as algorithm bias, data privacy, patient consent, and regulatory issues must be addressed beforehand [46]. The clinical importance of detecting OLNM correctly cannot be underestimated. At the present moment, in the management of cN0 OSCC, the choice should be made between the strategy of watchful waiting and elective neck dissection (END). There is enough evidence for performing END in patients who have more than a 20% chance of occult nodal disease [29]. AI-based risk scores will allow calculation of individual probabilities of occurrence of this condition much more accurately compared to the current clinical models, which are based on tumor DOI only [47]. As it was shown in the model of Farrokhian et al. [29], AI-based DOI-prediction systems can stratify patients into high- and low-risk groups much more accurately compared to existing T-staging and DOI criteria.

Future research should focus on the following areas: (1) prospective multicenter validation studies using a variety of patient samples; (2) comparative studies to test the efficacy of AI models against existing clinical staging systems; (3) standardized protocol of imaging and radiomic features' extraction; (4) the use of XAI systems; (5) assessment of the cost-effectiveness of AI-based neck management; and (6) ethical and regulatory aspects of the use of AI technology in the field of oral oncology. The development of federated learning methodologies, which provide for the protection of patients' privacy while training models across several institutions, is a very promising methodological approach. The development of lightweight DL algorithms, like the one used by Gang et al. [42], provides a way to implement AI in the context of LMICs. At the same time, multitask DL models, like the ones described by Lan et al. [41], allow for comprehensive oncological examination based on a single preoperative MRI [46].

## Conclusions

AI demonstrates substantial potential as a diagnostic adjunct for detecting OLNM in oral cancer. DL CNN architectures and hybrid radiomics-DL models achieve the highest diagnostic accuracy reported to date, substantially surpassing conventional imaging. Despite promising results, current evidence is limited by methodological heterogeneity, geographic concentration, small sample sizes, and lack of external validation. Rigorous prospective multicenter studies, algorithmic standardization, and ethical frameworks for clinical integration are required before widespread clinical adoption can be recommended.

## Appendices

**Table 4:** Studies assessed in full text but not meeting the eligibility criteria. SCC: squamous cell carcinoma; AI: artificial intelligence; LNM: lymph node metastasis; AUC: area under the curve; HNC: head and neck cancer; ENE: extranodal extension; HNSCC: head and neck squamous cell carcinoma; OSCC: oral squamous cell carcinoma; NSCLC: non-small cell lung cancer; ML: machine learning.

S. No.	Study (first author)	Journal & year	Reason to exclude (tailored to a radiomics/AI HNC prognosis/diagnosis review)	Typical exclusion criterion wording
1	Tao et al., 2015 [48]	Radiother Oncol, 2015	Evaluates atlas‑based auto‑segmentation and dosimetric consistency, not radiomics/AI linked to clinical outcomes.	“Studies focused solely on segmentation methodology or dosimetry without radiomic feature extraction linked to clinical endpoints.”
2	Kann et al., 2018 [49]	Sci Rep, 2018	Deep‑learning CT classification of nodal metastasis/ENE; not a radiomics feature–outcome study (and/or not primary‑tumor–based).	“Deep learning image‑classification studies without radiomic feature extraction and statistical modeling of clinical outcomes.”
3	Haarburger et al., 2021 [50]	Sci Rep, 2021	Technical reproducibility of radiomics features across lung/kidney/liver; no HNC cohort and no patient‑level clinical endpoints.	“Technical/reproducibility studies without patient‑level clinical endpoints; non-head‑and‑neck populations.”
4	Badic et al., 2019 [51]	Sci Rep, 2019	Radiogenomics in colorectal cancer; wrong disease site and not an HNC radiomics‑only prognostic model.	“Studies not involving head‑and‑neck cancer patients.”
5	Haider et al., 2020 [52]	Cancers Head Neck, 2020	Narrative/review of radiomics applications in HNSCC; no primary patient data or original outcomes.	“Reviews, editorials, commentaries, and non‑primary research.”
6	van Dijk et al., 2019 [53]	Sci Rep, 2019	Delta‑radiomics of parotids to predict late xerostomia (toxicity), not tumor diagnosis/prognosis/oncologic outcomes.	“Studies predicting treatment toxicity rather than tumor diagnosis, staging, or oncologic outcomes.”
7	Safi et al., 2017 [54]	J Craniomaxillofac Surg, 2017	Conventional clinicopathological risk‑factor analysis for OSCC recurrence; no imaging radiomics or AI.	“Studies not using radiomic features or machine/deep learning on medical images.”
8	Ger et al., 2019 [55]	PLoS One, 2019	HNC radiomics study showing no added value over simple clinical variables; may fail prespecified performance/validation thresholds.	“Studies where radiomics models did not improve prediction over baseline clinical models or lacked meaningful external validation.”
9	Parmar et al., 2015 [56]	Sci Rep, 2015	Methods/feature‑selection paper in NSCLC comparing ML pipelines; not an HNC disease‑specific clinical model.	“Methodological studies in non-head‑and‑neck cohorts without disease‑specific clinical model development/validation.”
10	Lin et al., 2019 [57]	Aging, 2019	Immunogenomic prognostic index in papillary thyroid cancer; wrong disease and non‑imaging modality.	“Non‑HNC populations and/or non‑imaging (genomic/immunogenomic) prognostic studies.”

**Table 5:** PRISMA 2020 compliance checklist. Items 10a, 10b, and 14 are new additions in PRISMA 2020. All 27 items are addressed in this manuscript.

Section and topic	Item	PRISMA 2020 Checklist Item	Location in manuscript
TITLE	1	Identify the report as a systematic review.	Title page
ABSTRACT	2	See PRISMA 2020 for Abstracts checklist.	Abstract
INTRODUCTION			
Rationale	3	Describe the rationale for the review in the context of existing knowledge.	Introduction
Objectives	4	Provide an explicit statement of the review objective(s) or question(s).	Introduction (last paragraph)
METHODS			
Eligibility criteria	5	Specify the inclusion and exclusion criteria and how studies were grouped for synthesis.	Section 2.3
Information sources	6	Specify all databases, registers, websites, organizations, reference lists, and other sources searched or consulted, including the date last searched.	Section 2.2
Search strategy	7	Present the full search strategy for all databases, including filters and limits used.	Section 2.2; Supplementary Material
Selection process	8	Specify the methods used to decide whether a study met the inclusion criteria, including the number of reviewers and independence of screening.	Section 2.4
Data collection process	9	Specify the methods used to collect data from reports, including the number of reviewers.	Section 2.5
Data items	10a	List and define all outcomes for which data were sought.	Section 2.5
Data items	10b	List and define all other variables for which data were sought.	Section 2.5
Risk of bias assessment	11	Specify the methods used to assess risk of bias, including the assessment tool.	Section 2.6
Effect measures	12	Specify the effect measures used for each outcome.	Section 2.7
Synthesis methods	13a–f	Describe the methods used to synthesise results, including rationale for not performing a meta-analysis.	Section 2.7
Reporting bias assessment	14	Describe methods used to assess risk of bias due to missing results.	Section 2.7
Certainty assessment	15	Describe methods used to assess certainty (or confidence) in the body of evidence.	Section 2.7
RESULTS			
Study selection	16a	Describe the search and selection process from records identified to studies included.	Section 3.1; Figure 1
Study selection	16b	Cite studies that appeared to meet inclusion criteria but were excluded and explain why.	Table 4
Study characteristics	17	Cite each included study and present its characteristics.	Table 1
Risk of bias in studies	18	Present assessments of risk of bias for each included study.	Table 2
Results of individual studies	19	Present summary statistics and effect estimates for each study.	Table 1
Results of syntheses	20a–d	Present results of all syntheses performed, including heterogeneity and certainty where applicable.	Results; Discussion
Reporting biases	21	Present assessments of risk of bias due to missing results for each synthesis.	Section 4.1
Certainty of evidence	22	Present assessments of certainty (or confidence) in the body of evidence.	Table 3
DISCUSSION			
Discussion	23a	Provide a general interpretation of the results.	Section 4
Discussion	23b	Discuss limitations of the evidence included in the review.	Section 4.1
Discussion	23c	Discuss limitations of the review processes used.	Section 4.1
Discussion	23d	Discuss implications for practice, policy, and future research.	Sections 4.2 and 4.3
OTHER INFORMATION			
Registration and protocol	24a	Provide registration information, including register name and registration number.	Section 2.1
Registration and protocol	24b	Indicate where the review protocol can be accessed or state if unavailable.	Section 2.1
Registration and protocol	24c	Describe amendments to information provided at registration or in the protocol.	Not applicable
Support	25	Describe sources of financial or non-financial support and the role of funders.	Declarations
Competing interests	26	Declare any competing interests of review authors.	Declarations
Availability of data, code and other materials	27	Report which materials are publicly available and where they can be found.	Declarations/Data Availability Statement
